# A Hidden Role of the Inactivated FANCD2: Upregulating ΔNp63

**DOI:** 10.18632/oncotarget.1217

**Published:** 2013-08-11

**Authors:** Jayabal Panneerselvam, Anna Pickering, Jun Zhang, Hong Wang, Hui Tian, Junnian Zheng, Peiwen Fei

**Affiliations:** ^1^ University of Hawaii Cancer Center, University of Hawaii, Honolulu, HI, USA; ^2^ Department of Laboratory Medicine and Pathology, Mayo Clinic, Rochester, MN, USA; ^3^ Jiangsu Key Laboratory of Biological Cancer Therapy, Xuzhou Medical College, China; ^4^ present address: Department of Gastroenterology; First Municipal People's Hospital of Guangzhou; First Affiliated Hospital Sun Yat-Sen University; Guangzhou, China

**Keywords:** P63, Fanconi Anemia tumor supressor pathway, oncogene delta N p63, FANCD2, tumorigenicity, transactivation

## Abstract

A compromised Fanconi Anemia (FA) signaling pathway, often resulting from an inactivated FANCD2, was recently recognized to contribute to the development of non-FA human tumors. However, it is largely unknown as to how an impaired FA pathway or an inactivated FANCD2 promotes tumorigenesis. Here we unexpectedly found that ΔNp63 mRNA was expressed at high levels in human cancer cells carrying an impaired FA pathway compared to the corresponding control cells carrying an intact FA pathway. This observation was recapitulated upon conditionally managing the status of FANCD2 monoubiquitination /activation in 293T cells. Importantly, ΔNp63 elevation upon FANCD2 inactivation was confirmed in human fibroblasts derived from FA patients. Moreover, we have identified a 189 bp DNA fragment downstream of the ΔNp63 promoter (P2) that can mediate the upregulation of ΔNp63 by an inactivated FANCD2, and determined that elevated ΔNp63 is high enough to promote cancer cell proliferation and metastasis. *In vivo*, the elevation of FAVL, a tumor promotion factor that inhibits FANCD2 activation, was found to be positively associated with ΔNp63 expression in human cancer tissues. Collectively, these results document a novel role of an inactivated FANCD2 in upregulating ΔNp63, advancing our understanding of how an impaired FA pathway contributes to the pathogenesis of human cancer.

## INTRODUCTION

The alternative promoter (P2) of p63 leads to deleted-transactivation domain (TA) isoforms (ΔNp63), while transcription starting at the P1 promoter of p63 produces TA-containing p63 isoforms [1-4]. P63-containing the transactivation domain is capable of inducing apoptosis and inhibiting cell-cycle progression, thus suppressing tumor development [1-3, 5-7]. On the other hand, a ΔNp63 isoform resulting from the alternative P2 promoter generally enhances proliferation and inhibits apoptosis, and thus promotes tumorigenesis [2, 3, 8]. The ΔNp63 variants are often overexpressed in a variety of human cancers, including squamous cell origin (head and neck, lung), breast and bladder [8]. In head and neck squamous cell carcinoma and “triple-negative” breast cancer cells, ΔNp63 suppresses p73-dependent apoptosis and thus promotes tumor survival, and its expression correlates with poor prognosis of cancer patients [9-12].

Fanconi Anemia (FA) is a cancer-prone, rare human genetic disease, resulting from mutations in a group of genes that encode products known to function in one common DNA damage response pathway called the FA or FA-BRCA pathway [13-16]. The improper function / transduction of the FA pathway confers the defects in repairing damaged DNA, especially DNA crosslinks, and ultimately leads to chromosome instability and the development of both FA and non-FA human tumors [15, 17-20]. FANCD2 monoubiquitination is a hallmark of the activation of the FA pathway, and un-monoubiquitinated/inactivated FANCD2 accounts for nearly 95% of FA cases, and is one of the major factors accounting for the tumorigenecity of an impaired FA pathway [14, 15, 21]. We are the first to demonstrate that an inactivated FANCD2, resulting from an FAVL-impaired FA complex E3 ubiquitin ligase, plays an important role in the development of non-FA human tumors [17, 18]. However, it remains largely unknown as to the mechanisms by which the inactivated FANCD2 leads to tumorigenesis. In this study, we found an unrecognized role of the inactive state of FANCD2 in the upregulation of ΔNp63 expression, indicating that the tumorigenicity of an impaired FA pathway is partly attributed to the subsequently elevated ΔNp63.

### RESULTS

#### ΔNp63 Appears to Be a Downstream Target of Inactivated FANCD2

**Figure 1:**
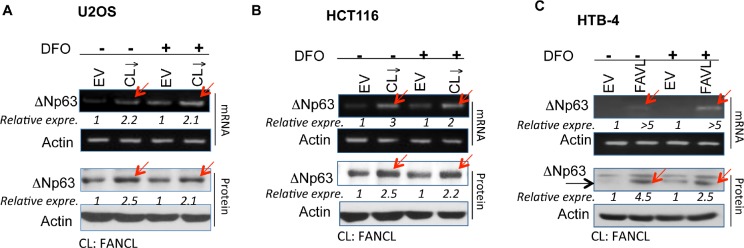
ΔNp63 expression is elevated in cancer cells carrying an inactivated FA pathway/ inactivated FANCD2 under normoxic and hypoxic conditions Sets of HCT116, U2OS, and HTB-4 stably-transfected cell pairs isogenic to the status of the FA pathway were used to detect ΔNp63 mRNA and protein levels. Levels of both ΔNp63 mRNA and protein are higher in cells carrying an impaired FA pathway compared to the corresponding empty vector-containing control cells (carrying an intact FA pathway) under normal growth conditions or the treatment with 280 μM of Deferoxamine (DFO, a hypoxia-mimicking drug). (A) HCT116 colon cancer and (B) Human osteosarcoma U2OS cells stably express a down-regulated FANCL (Supplementary Figure 1). (C) HTB-4 bladder cancer cells stably express a higher level of FAVL and carry an impaired FA pathway [18]. *(NIH Image J software was used to evaluate the band densities, with which the ΔNp63 expression levels were calculated upon the corresponding control =1)*.

ΔNp63 is often highly expressed in a variety of human cancers, including squamous cell origin (head and neck, lung), breast and bladder cancers [8]. Coincidently, there is a high incidence of a variety of human cancers associated with FA patients, especially head and neck cancers [22]. We therefore asked whether an impaired FA signaling pathway has any relation to the high expression levels of ΔNp63. We generated two sets of stably-transfected cell pairs (Supplementary Figure 1), isogenic to the level of FANCL expression that would result in an impaired or intact status of the FA pathway [23], to examine how the status of the FA pathway is relevant to the level of ΔNp63 expression. Surprisingly, under both normal and hypoxic growth conditions (the latter is induced by a hypoxic mimicking drug), ΔNp63 mRNA and protein expression levels were found to be elevated in U2OS and HCT116 cells carrying an impaired FA pathway as compared to the corresponding empty vector-transfected control cells, in which the FA pathway is intact (Figures 1A and B). To confirm that ΔNp63 elevation results from an inactivated FANCD2, not from the off-target effect of FANCL silencing, we detected ΔNp63 expression in stably-transfected cell pairs derived from the HTB-4 bladder cancer cell line, in which the impaired FA pathway was induced by a high expression level of FAVL (a variant of FANCL that acts as a tumor promotion factor by inactivating FANCD2) [17, 18]. We found that HTB-4 cells carrying an inactivated FANCD2 do express ΔNp63 at a higher level in comparison with the corresponding control HTB-4 cells (Figure 1C). Together, ΔNp63 appears to be a downstream target of inactivated FANCD2 not only under the normal cell growth condition but also under conditions of hypoxia. This suggests that the regulation of ΔNp63 expression by inactivated FANCD2 may play an important role in the development of human tumors, presumably starting from tumor initiation to tumor mass development, which often leads to hypoxic conditions within solid tumors.

#### ΔNp63 Elevation can Be Recapitulated in Human Non-cancer Cells, Including FA Patient Cells, upon Altering the Status of FANCD2 Monoubiquitination

**Figure 2:**
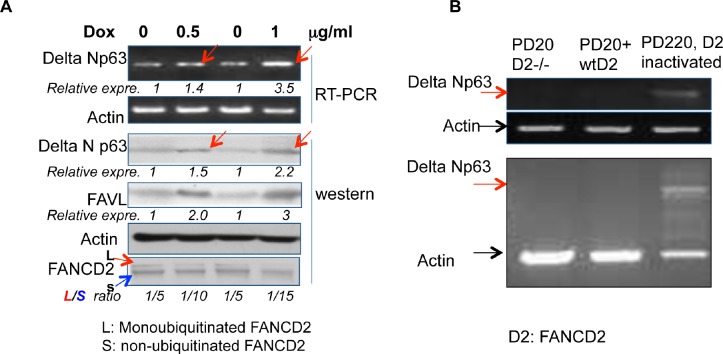
ΔNp63 elevation is recapitulated in 293T cells and FA patient cells upon the inactivated FANCD2 (A) A higher level of ΔNp63 expression follows FAVL elevation in a Tet-on inducible expression system. The 293T conditional expression cell line was established to express FAVL controlled by the inducer, Doxycycline (Dox). 0.5 or 1 μg/ml Dox addition can induce FAVL expression at a higher level than non-treated cells (0 μg/ml Dox). Under the same conditions, the levels of ΔNp63 expression are elevated and the basal levels of FANCD2 monoubiquitination are reduced (monoubiquitinated/non-ubiquitinated ratio is decreased). The conditional overexpression of FAVL that provides an impaired FA pathway was correlated with the corresponding elevation of ΔNp63 expression *(NIH Image J software was used to evaluate the band densities, with which the relative FAVL and ΔNp63 expression levels were calculated upon the corresponding control =1 for non-induced cells*. monoubiquitinated/non-ubiquitinated *ratio was generated also upon the band densities)*. (B) ΔNp63 mRNA expression is only detectable in PD220 cells (FANCA−/−), but not in PD20 (FANCD2−/−) and PD20+FANCD2 cells. Total RNA was isolated from normally growing PD220, PD20 and PD20+FANCD2 cell. The expression of ΔNp63 mRNA was detected by RT-PCR, which was clearly detectable in PD220 (FANCA−/−), and not in PD20 (FANCD2−/−) or PD20+FANCD2 cells. The un-monoubiquitinated FANCD2, but not the loss of FANCD2, is able to elevate ΔNp63 expression. *[Two of three separated RT-PCR results are shown, without (top panel) or with (bottom panel) control actin amplification in the same PCR reaction]*.

To validate the relationship between an impaired status of the FA pathway and the expression levels of ΔNp63, we tested the association between the levels of ΔNp63 expression and an impaired FA status in non-tumorigenic 293T cells wherein the genetic background is relatively closer to normal cells as compared to the tested tumor cells above (HCT116, U2OS and HTB-4). We generated Tet-on inducible 293T stably-transfected cells, within which FANCD2 is only inactivated when FAVL is overexpressed, controlled by the conditional inducer, Doxycycline (Dox, a more stable tetracycline analogue). We found that the level of ΔNp63 expression is correspondingly elevated when the level of FAVL expression is increased (Figure 2A). This observation confirms the above finding (Figure 1), indicating that the regulation of ΔNp63 expression by inactivated FANCD2 is not restricted to tumor cells. We further validated the association of ΔNp63 expression with inactivated FANCD2 by using FA patient cells, in which the variables only result from the FANCD2 status. We examined the level of ΔNp63 mRNA expression in PD20 (FANCD2−/−), PD20+FANCD2, and PD220 (FANCA−/−; carrying an unstable E3 ubiquitin ligase complex, leading to an un-monoubiquitinated FANCD2). We found that the ΔNp63 mRNA expression level is only detectable in PD220 cells in which FANCD2 is inactivated, but not in PD20 cells with or without a reconstituted wtFANCD2 (Figure 2B). Collectively, ΔNp63 elevation is associated with an impaired status of the FA pathway, and it may act as a tumorigenic mediator of inactivated FANCD2 during tumor development.

#### Inactivated FANCD2 Employs the 1.2 kb DNA Fragment Downstream of the P2 Promoter to Regulate ΔNp63 Transcription

**Figure 3:**
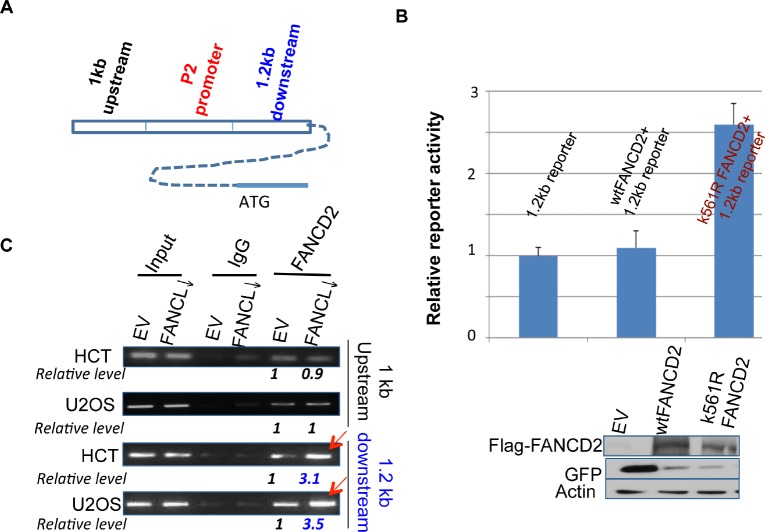
Inactivated FANCD2 promotes the expression of ΔNp63 via a 1.2 kb DNA fragment downstream of the P2 promoter (A) Schematic representation of 1 kb and 1.2 kb DNA fragments up or downstream of the P2 promoter. (B) Inactivated FANCD2 enhances the 1.2 kb reporter activity. Both the 1.0 kb and 1.2 kb DNA fragments (Fig. 3A) were individually cloned into the upstream of pGL-3-promoter- reporter, named 1 kb or 1.2 kb reporters. Cells showed a higher reporter activity when the 1.2kb reporter was co-transfected with K561R FANCD2 cDNA-containing plasmid. The relative reporter activity was plotted upon photon counts as we did previously. Cells carrying the 1.0kb reporter along with either wt or mtFANCD2 cDNA did not show a noticeable difference in the reporter activity as compared to the 1.0 kb reporter alone (not shown). The results shown are a representative of five independent experiments performed each time in triplicate, and error bars indicate the standard deviation. The transfection efficiency of the reporter assay for pEGFP-Flag-wtFANCD2 or -mtFANCD2 was measured via western blotting analysis with antibodies against GFP (the pEGFP vector produces polycistronic mRNAs encoding non-fusion GFP protein) and Flag-fused FANCD2 protein. (C) The inactivated FANCD2 associates more strongly with the 1.2 kb DNA fragment. Both HCT116 and U2OS sets of stably-transfected cell pairs carrying an intact or impaired FA pathway, respectively (Supplementary Figures 1 and 3 right panel) were used to perform FANCD2 ChIP analysis using primers to bracket DNA fragments 1 kb up or 1.2 kb downstream of the P2 promoter (Figure 3A). (*The relative folds were calculated upon the band density measured by NIH image J program with the corresponding bands generated from control cells as “1”.)*

To define the association between inactivated FANCD2 and an enhanced level of ΔNp63 expression, we asked whether inactivated FANCD2 plays a direct role in the regulation of ΔNp63 mRNA expression. We constructed a ΔNp63 promoter (P2)-containing luciferase reporter, and co-transfected the reporter with wt or K561R FANCD2 cDNA-containing plasmids (K561R FANCD2 cDNA encodes a FANCD2 protein lacking the lysine residue required for monoubiquitination). We did not observe any change in reporter activity (data not shown) to support the above finding (Figures 1 and 2). We also performed chromatin immunoprecipitation (ChIP), which did not show an interaction between the P2 promoter and mtFANCD2 nor wtFANCD2 (data not shown). Considering enhancers that can promote transcription, we used the sequences 1 kb upstream and 1.2 kb downstream of the P2 promoter to construct two new reporters respectively (Figure 3A). Through the reporter assay, we found that cells carrying mtFANCD(K561R)-containing plasmid showed a higher luciferase activity when co-transfected with the reporter containing the 1.2 kb DNA fragment downstream of P2 as compared to the cells transfected with empty vector or wtFANCD2 in various combinations, all of which showed a similar basal level of luciferase activity (Figure 3B; data not shown). These results suggest that inactivated FANCD2 may play a role in enhancing the transcription of ΔNp63, which appears to be a new function for the inactivated FANCD2, rather than a loss function of wtFANCD2. To support the reporter activity observed, we conducted ChIP analysis on the binding potential of inactivated FANCD2 protein to the 1.2 kb DNA fragment. We found that antibodies against inactivated FANCD2 can also pull down a substantial amount of the downstream DNA fragment, but not the one upstream of the P2 promoter (Figure 3C), which agrees with the reporter assay (Figure 3B). Therefore, inactivated FANCD2 can regulate ΔNp63 mRNA expression through the association with a DNA sequence downstream of the known P2 promoter of ΔNp63.

#### Identification of a 189 bp DNA Fragment Which Mediates the Regulation of ΔNp63 Expression by an Inactivated FANCD2

**Figure 4:**
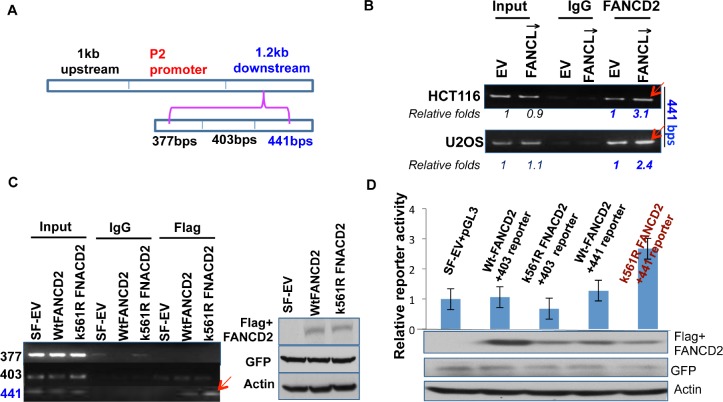
A 441bp DNA sequence within the 1.2kb fragemtn can mediate the regulation of ΔNp63 expression by inactivated FANCD2 (A) The 1.2 kb DNA fragment was divided into three DNA segments with a size of 377, 401, or 441 bps. (B) 441 bp DNA fragment, but not the 377 and 403 bp ones (Supplementary Figure 2, left panel), can be pulled down more by FANCD2 antibodies in U2OS and HCT116 stably-transfected cells carrying an inactivated FANCD2 compared to corresponding control cells with an intact FA pathway. (C) the 441 bp DNA fragment can also be pulled down more by Flag antibodies in cells transiently co-transfected with Flag-mtFANCD2, but not Flag-wtFANCD2. A ChIP assay was performed in cells transiently transfected with pEGFP-Flag wtFANCD2 or mtFANCD2 by using Flag antibodies. ChIP-PCR primers were designed for three fragments shown in (A). The similar transfection efficiency was measured through western blotting by using antibodies against GFP or Flag (right panel). (D) K561R mtFANCD2 stimulates the 441-reporter activity. Similarly as previously done, three DNA segments were individually cloned into the upstream of pGL-3-promoter-reporter, and named the 377, 403, or 441 -reporters. 293T cells were transiently transfected respectively with these reporters and pEGFP-Flag plasmids containing wt or mtFNACD_2_. All luciferase assays were normalized for transfection efficiency by renilla reporter activity. Western blot analysis with Flag or GFP antibodies was performed to verify expressions of both wt and mtFANCD2 proteins. The results shown are a representative of five independent experiments performed each time in triplicate, error bars indicating the standard deviation [the 377-reporter (not shown) produces luciferase activity similar to the one derived from the 403-reporter].

**Figure 5:**
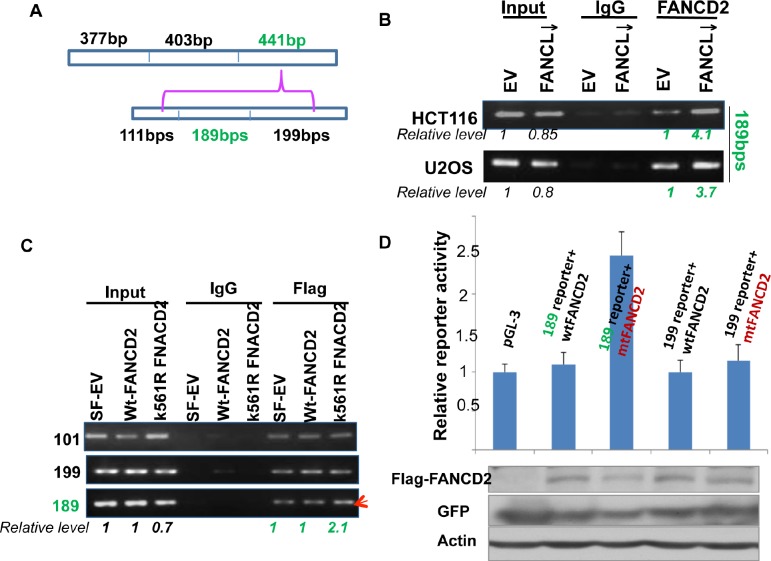
A 189bp DNA sequence within the 441 bp segment can mediate the regulation of ΔNp63 expression by mtFANCD2 (A) The 441 bp DNA fragment was divided into three parts with sizes of 111, 189, and 199 bps. (B) Both HCT116 and U2OS stably-transfected cell pairs, carrying an intact or impaired FA pathway, were used for the ChIP assay with FANCD2 antibodies. The 189 bp, but not 111 or 199 bp DNA fragments (Supplementary Figure 2, left panel) was pulled down more by FANCD2 antibodies from lysates prepared form cells carrying an activated FANCD2 compared to the corresponding control cells carrying an intact FA pathway *(Relative levels were calculated upon the band intensity measured via NIH image J software)*. (C) The 189bp DNA fragments can be pulled down more by antibodies specifically targeting mtFANCD2. The template DNA for ChIP-PCR was shared with that performed for Figure 4C. (D) Cells carrying the 189-reporter show a higher luciferase activity when co-transfected with pGEFP-Flag-mtFANCD2, but not pGEFP-Flag-wtFANCD2. Similarly as previously done, three DNA segments were individually cloned into the upstream of pGL-3-promoter-reporter, named the 101, 189, or 199-reporters. 293T cells were transiently transfected with these pGL-3 derivative reporter plasmids containing the 101, 189, or 199 bp DNA fragments and pEGFP-Flag plasmids encoding wt or mtFNACD2. All luciferase assays were normalized for transfection efficiency with a renilla reporter. Western blot analysis with Flag or GFP antibodies was performed to verify protein expression levels of wt or mtFANCD2 (shown at the bottom panel). The results shown are a representative of five independent experiments performed each time in triplicate, error bars indicating the standard deviation [the 101-reporter (not shown) carries a similar activity to the 199-reporter].

Next, we wanted to narrow down the specific region within the 1.2 kb DNA fragment that mediates the transactivation activity of the inactivated FANCD2 (Figures 1-3). By dividing the 1.2 kb DNA fragment into three segments with a size of about 400 bp each (Figure 4A), we designed three sets of primers and performed ChIP assays by using U2OS and HCT116 stably-transfected cell pairs carrying activatible or in-activatible FANCD2. We found that the third 441 bp DNA fragment (Figure 4A) can be pulled down more along with the FANCD2 protein from the lysates prepared from cells carrying in-activatible FANCD2 (Figure 4B; Supplementary Figure 2). This was further validated in 293T cells transiently transfected with Flag-wt or mtFANCD2 plasmids. As shown in Figure 3C, a greater pull-down of the 441 bp fragment by Flag antibodies was seen in the lysates prepared from 293T cells containing Flag-mtFANCD2 compared to the same cells transfected with other reporters. Correspondingly, we generated three reporter constructs to have an approximately 400 bp DNA fragment located upstream of the SV40 promoter of the pGL-3 luciferase reporter. We found the third fragment-containing reporter (441-reporter) had a higher luciferase activity in cells transfected with mtFANCD2 compared to cells transfected either with wtFANCD2 or the empty vector control. These reporters appeared to have a similar level of luciferase activity, suggesting again that the enhanced reporter activity is initiated by mtFANCD2, but not the loss function of wtFANCD2 (Figure 4D and data not shown). Similarly, we further divided the 441bp DNA fragment (Figure 5A) and found a DNA fragment with a size of 189 bps (Figures 5B-D; Supplementary Figure 2), which can mediate the enhanced reporter activity initiated by inactivated FANCD2 as well as the *in vivo* association with inactivated FANCD2. Taken together, the 189 bp DNA fragment downstream of the P2 promoter is a *cis*-element that can mediate the upregulation of ΔNp63 expression by inactivated FANCD2 at the genetic level.

#### ΔNp63 is a Potent Mediator for an Impaired FA Pathway in Promoting Tumorigenesis

**Figure 6:**
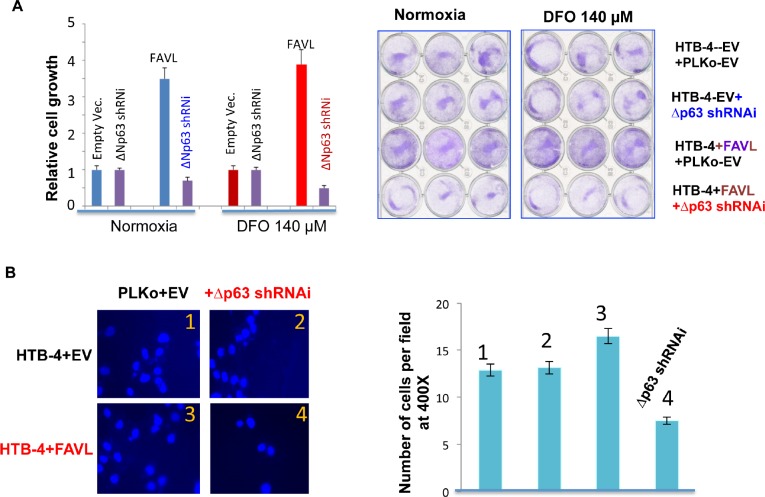
Silencing ΔNp63 mRNA expression substantially abrogates the tumorigenic potential of HTB-4 bladder cancer cells triggered by FA pathway impaired by elevated FAVL (A) Silencing ΔNp63 blocks the advantage in cell proliferation triggered by FAVL elevation under both hypoxia and normoxia (normal oxygen). HTB-4+EV or +FAVL stably-transfected cells with or without silenced ΔNp63 (Supplementary Figure 3) were plated at day 0 with an equal number. The total cell number was recounted on day 5 and plotted in the left panel for the relative cell growth. The representative images on day 5 were shown for cells growing under both normoxia and hypoxic stress (right panel). (B) Silencing ΔNp63 blocks the invasive advantage triggered by FAVL elevation. As performed previously [11], two sets of stably-transfected cell pairs (HTB-4+EV+Lenti-EV & HTB-4+EV+Lenti-shRNAi; HTB-4+FAVL+Lenti+EV&HTB-4+FAVL+Lenti-shRNAi) (Supplementary Figure 3) were grown in 140 μm DFO overnight and plated into transwells the following day in medium containing 140 μm DFO. Transwells were processed accordingly 2 hours after plating. Cells were counted with 10 random fields at a magnification of 400X. All counted cell numbers were used to plot the relative invasive potential (right panel). Representative images of the invasive cell density are shown in the left panel. Cells expressing elevated FAVL have a stronger invasive potential (image panel-3 compared to image panel-1). Silencing ΔNp63 can mitigate the invasive potential of cells carrying FAVL (image panel 4, compared to image panel 3), but not cells without FAVL elevation (image panel 2 compared to image panel-1; SD was made from three separate experiments).

**Figure 7:**
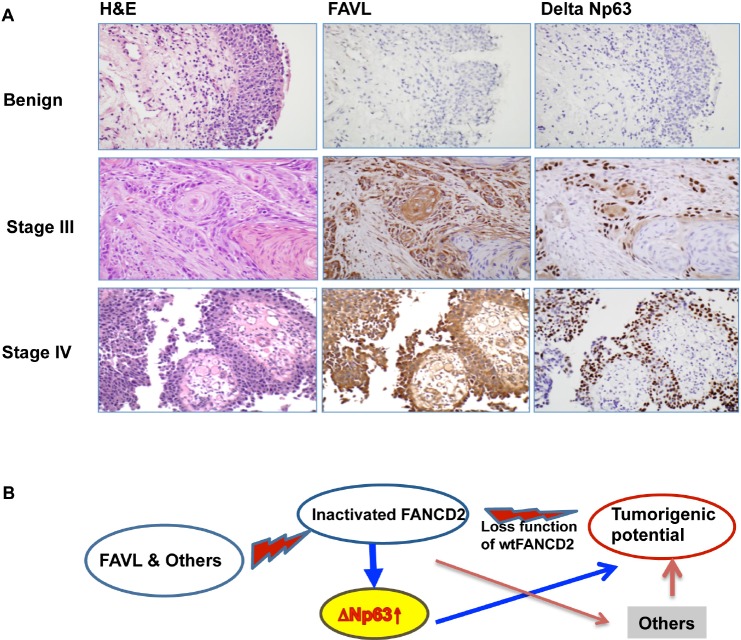
ΔNp63 can mediate tumorigenecity of an impaired FA pathway/an inactivated FANCD2 (A) FAVL expression positively correlates with ΔNp63 expression *in vivo*. A total of 25 human bladder cancer samples were used to detect the levels of FAVL and ΔNp63 protein expression *in situ* via immunohistochemistry (the staining intensity was summarized in Supplementary Table 1). Three sets of representative images are shown to indicate the *in situ* association accompanying specific tumor stages, determined by H&E staining (*All used slides for each individual case were made from consecutive tissue slides, which reserve the same tissue landscape)*. (B) A proposed working model. FAVL and others can inactivate FANCD2 and lead to an impaired status of the FA pathway, which in turn promotes tumorigenesis. Inactivated FANCD2 not only loses the function that an activated FANCD2 performs but also gains new functions including upregulating ΔNp63, together contributing to the development of both FA and non-FA human cancers.

Our study thus far reveals that the regulation of ΔNp63 expression by an inactivated FANCD2 may play important roles in the tumor promotion potential resulting from an impaired FA pathway. Whether the gained growth potential of cells harboring an impaired FA pathway is partly attributed to ΔNp63 needs to be tested. We examined the growth potential of FAVL-elevated HTB-4 cells with or without silencing ΔNp63 expression (Supplementary Figure 3). As shown in Figure 6A, the gained growth potential resulting from the elevated FAVL can be mitigated by silencing ΔNp63 expression, but there is no noticeable difference between empty vector-containing HTB-4 cells with or without silenced ΔNp63. Furthermore, the metastatic potential resulting from FAVL elevation also shows a similar pattern of change (Figure 6B). These results demonstrate that inactivated FANCD2 is not only able to upregulate ΔNp63 expression, but also that elevated ΔNp63 expression can contribute to tumor development through, at least, enhancing the potentials of cell proliferation and metastasis. We next explored the clinical importance of upregulation of ΔNp63 by inactivated FANCD2. Using immunohistochemistry, we found the level of FAVL expression (cytoplasmic staining) is positively associated with the level of ΔNp63 protein expression (nuclear staining) in 25 human bladder tissue samples tested (Figure 7A; Supplementary table 1). Taken together, both *in vitro* and *in vivo* data reveal that ΔNp63 is a functional mediator of an impaired FA pathway triggered by FANCD2 inactivation, but not by the loss of activated FANCD2. This indicates that the tumorigenicity of inactivated FANCD2 in human tumors, such as human bladder cancer, is at least partly attributed to the subsequent elevation of ΔNp63 (Figure 7B).

### DISCUSSION

The p63 gene, together with p73 and p53, belongs to the p53 gene family, all of which play crucial rols in tumor supression [24-28]. Although these gene products show common structural and functional features, each protein appears to have specific biological functions. p53-deficient mice grow normally but undergo spontaneous tumor development, while p73 and p63 knockout mice do not develop tumors but exhibit developmental and differentiation defects [29, 30]. The p63 gene generates the expression of two subclasses of isoforms, namely, those containing the TA, called TA isoforms, and those lacking this domain, called ΔN isoforms [31]. Accumulated studies indicate that ΔNp63 is a tumor promotion factor [8-10], because these ΔN isoforms are often overexpressed in human tumors, including human bladder cancer; especially, ΔNp63 can antagonize apoptosis.

Here we have shown that inactivated FANCD2 can upregulate ΔNp63 expression substantially, which provides a novel understanding of the roles of compromised FA signaling in human tumorigenesis. Utilizing a series of ChIP and reporter assays we found that a DNA fragment with a size of 189 bps downstream of the alternative promoter (P2) of the p63 gene (Figures 3-5), is a potent *cis*-element responsible for the regulation of ΔNp63 expression by inactivated FANCD2. To this point, it is unclear whether this specific DNA fragment directly interacts with inactivated FANCD2. Nonetheless, both ChIP and reporter assays demonstrate that inactivated FANCD2 can substantially upregulate ΔNp63 through the 189 bp DNA segment downstream of the P2 promoter as compared to wtFANCD2, which showed a minimal effect on the regulation of ΔNp63 expression, essentially equivalent to the control. This indicates that the tumorigenicity of compromised FANCD2 activation [13, 17] is, at least partly, attributed to inactivated FANCD2-triggerred ΔNp63 expression, and thus provides a novel insight into the development of an effective tool for genetically intervening in cancer cell growth.

The strong association among a lack of DNA damage repair, mutations and cancer is dramatically demonstrated by a number of cancer susceptibility syndromes [32,33], including FA [22]. Accumulated studies strongly suggest the tumor suppressor activity of the FA signaling pathway is derived from its functional aspect at DNA damage repair [14, 18, 34]. Therefore, the monoubiquitinated/activated FANCD2, a center of the FA signaling pathway, becomes essential in DNA damage repair. Inactivated FANCD2, on the other hand, will lose the capability of repairing damaged DNA and lead to tumorigenesis. Our study reveals a previously unrealized role for inactivated FANCD2 in upregulating ΔNp63, contributing to the cancer susceptibility of FA as well as the tumorigenicity of an impaired FA pathway in the development of non-FA human tumors. A high level of ΔNp63 expression can be found in both malignant and non-malignant cells upon the alteration of the FA pathway (Figures 1 and 2). Also, expression levels of ΔNp63 and FAVL are positively correlated to each other *in vitro* and *in vivo* (Figures 1, 2 and 7A). Furthermore, downregulating ΔNp63 expression can mitigate the tumor cell growth and metastatic potential derived from an impaired FA pathway (Figure 6). These results document that ΔNp63 can act as a new mediator of inactivated FANCD2 that has the multifaceted effects on promoting human tumorigenesis, including the loss of the activated FANCD2's functions as well as the new function(s) gained (Figure 7B). In addition, our studies and those of others [14,17,18,35-38] have indicated that an impaired FA pathway genetically contributes to platinum-related drug sensitivity *in vitro*. Tumor cells, however, are sensitive initially to platinum-related chemotherapies in clinic, but develop drug resistance later over the course of treatment. We believe ΔNp63 elevation subsequent to FANCD2 inactivation may be an important factor that leads to drug resistance [39]. In the future, relevant translational studies, similar to many of those reported [40-43], will be able to shed light on the development of an effective tool to help conquer drug resistance.

## MATERIALS AND METHODS

### Cell lines, antibodies, chemicals, and RNAi oligos

All cell lines used were purchased from ATCC. The anti-FANCD2 antibody was purchased from NOVUS (cat#N100-182). The anti-ΔNp63 antibody was purchased from Santa-Cruz (cat#8172). The anti-Flag (cat# F3165) and anti-beta-actin (cat# 5316) antibodies were bought from Sigma. The cDNA sequence encoding ΔNp63 shRNAi is“CCGGTGCCCAGACTCAATTTAGTCTCGAGACTAAATTGAGTCTGGGCATTTTTG” and all ChIP PCR primers were synthesized by Invitrogen

### Immunohistochemistry (IHC), immunoblotting and quantitative RT-PCR

All methods used were described in our previous studies [16-18,35,44]. IHC was performed by using the FAVL antibodies with a 1:50 dilution ratio and the ΔNp63 antibodies with 1:200 dilution ratio for primary incubation, and followed by using the ImmPRESS Reagent Kit (Vector cat#MP-7401). For immunoblotting, both antibodies were used at a dilution ratio of 1:500 for primary incubation.

### Transfection

U2OS, HCT116, 293T and HTB-4 cells were cultured in DMEM containing 10% FBS. Transient transfections of plasmids were performed using Lipfectamine 2000 according to the procedures provided by the manufacturer.

### Reporter assays

293T cells were transiently transfected with pGL-3 reporter plasmids containing different fragments derived from the ΔNp63 promoter region and pGEFP-Flag plasmids encoding wt or mtFANCD2 together with the renilla reporter vector. For the luciferase activity assay, renilla and firefly luciferase activities were measured using the Dual-Luciferase kit (Promega, USA) according to the manufacturer's instructions. All luciferase assays were normalized for transfection efficiency with a renilla reporter vector. The results shown are a representative of five independent experiments performed each time in triplicate.

### Cell proliferation assay, Cell migration assay, and ChIP assays

As previously done [17, 18], invasive cells were counted from 10 fields randomly picked under a magnification of 400X for the cell migration assay. The primers used for ChIP-PCR are as follows:

**Upstream of the P2 promoter:**

F-5'-GACTTCGTGAAAGGTGAA-3'; R-5'-ATCTATGTAAATGATTAGTGG-3'.

**Downstream of the P2 promoter:**

F-5-GTAGGAGATGAAACAGTAGG-3'; R-5'-CAGGTATGACATGATGGACA-3'
377 bps: F-5'-GTAGGAGATGAAACAGTAGGAG-3', R-5'-AAGTCACCCTCCAGACGACA-3';
403 bps: F-5'-CTGTCGTCTGGAGGGTGAC-3', R-5'-TCCAAGCACTCACCTGCAAG-3';
441 bps: F-5'-CTTGCAGGTGAGTGCTTGGA-3', R-5'-CTCAGGTATGACATGATGGACAG-3';
111 bps: F-5'-CTTGCAGGTGAGTGCTTGGA-3', R-GCTCTACTCTTCCTCTTGCGG-3';
189 bps: F-5'-CAGACCGCAAGAGGAAGAGT-3', -R-TCTACTGAGGACAAGCAAGCC-3';
199 bps: F-5'-AACATTCAAGCAGGCTTGCTTGTC-3', R-CTCAGGTATGACATGATGGACAG-3'.

The PCR products were quantified by densitometry analysis using NIH image J software. All ChIP data were normalized with the corresponding input.

### Biospecimens

All biospecimens used in this study were under an approved IRBe protocol.

## Supplementary Figures and Table


