# Supplementary material for: A Hidden Role of the Inactivated FANCD2: Upregulating ΔNp63
Source: Oncotarget. 2013 Aug 11;4(9):1416–26. doi: 10.18632/oncotarget.1217 (PMC3824532; doi:10.18632/oncotarget.1217)
Supplement: Supplementary file 1 [file oncotarget-04-1416-s001.pdf]

Supplementary Figure 1.

The Establishment of HCT116 and U2OS stable cell pairs expressing FANCL at different levels

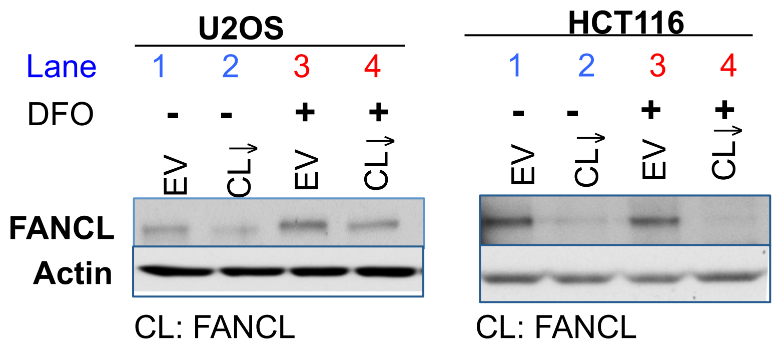

Fig S-1. Both HCT116 and U2OS cells were infected with lentivirus containing shRNAi targeting FANCL, a catalytic subunit of the FA complex E3. Cells were pool-selected with 2 μg/ml purimycin and verified by Western Blotting for the expression level of FANCL. The lanes (labeled with CL /FANCL down) show a clear low level of FANCL protein compared to corresponding empty vector-infected control cells (labeled with EV) under both normaxia (lanes 1 and 2) and hypoxia (lanes 3 and 4) (DFO is a hypoxic mimicking drug).

Supplementary Figure 2.

Four additional DNA segments studied associate with FANCD2 at different states with a similar affinity

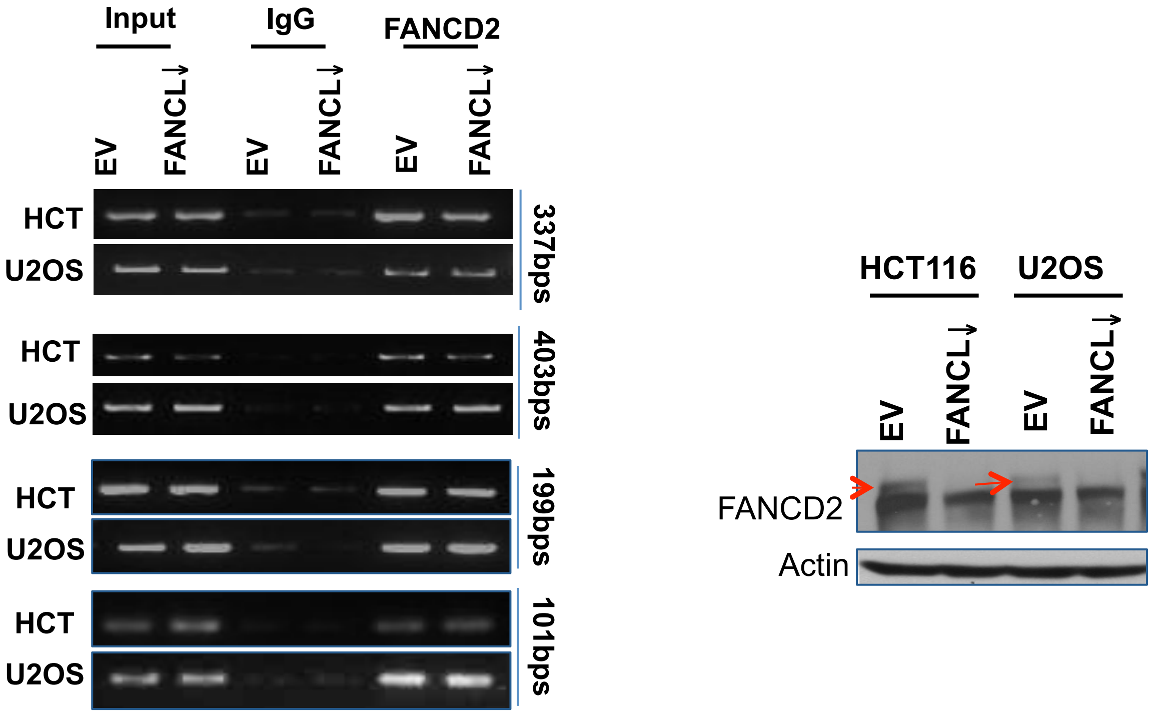

Fig S-2. Reduced monoubiquitination of FANCD2 at the basal level does not show a difference in the in vivo binding to the DNA fragments studied compared to the cells carrying a normal basal level of FANCD2 monoubiquitination. FANCD2 CHIP assay was performed by using anti-FANCD2 antibodies. FANCD2 pulled down a similar amount of DNA fragments down stream to P2 promoter in each set of stable cell pairs expressing FANCL at different levels. (40 cycles for CHIP PCR. Rabbit IgG used as the ChIP control).

Right panel: the basal level of FANCD2 monoubiquitination is reduced in FANCL-downregulated HCT and U2OS cells, which were used for CHIP assays shown in the left and Figures 4 and 5, as well as in Figure 1 for examining delta Np63 expression.

(→ Red arrowhead indicates the ubiquitinated FANCD2 at the basal level.)

Supplementary Figure 3.

The Establishment of stable cell pairs using HTB-4 derivative cell lines, expressing delta Np63 at different levels

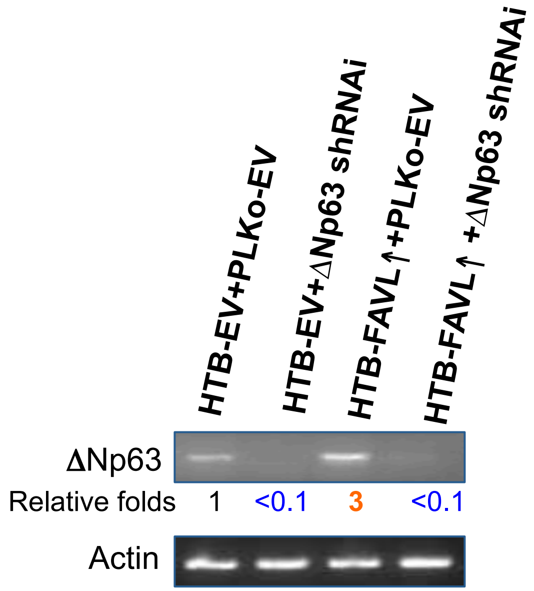

Fig S-3. Two HTB-4 derivative cell lines (HTB-4-EV and HTB-4+FAVL, isogenic to the level of FAVL expression, established in our previous study [11]) were used to generate two sets of stable cell pairs by infection with lentivirus-containing shRNAi to specifically target delta N p63. The level of delta Np63 mRNA was examined by RT-PCR, which shows a clear down-regulation of delta Np63 mRNA in both HTB-4-EV and HTB-4+FAVL cells. In addition, in HTB-4+FAVL cells, the level of delta N p63 mRNA is about 3 folds of the one shown in HTB-4-EV cells, indicating the FAVL elevation enhances the expression of delta Np63.

(40 cycles were done for PCR, instead of 35 cycles for all PCR reactions performed in the study.)

Supplementary Table 1

FAVL elevation associates with delta Np63 expression and tumor stages

Tumor stage & No of cases (25)	Benign 4 cases	Stage I 6 cases	Stage II 7 cases	Stage III & up 8 cases
FAVL expression	Not detectable	<+	+ to ++	++ up
ΔNp63 expression	Not detectable	Not detectable	+	+ up

+ indicating the relative staining intensity
